# Peripheral inflammation is associated with brain atrophy and cognitive decline linked to mild cognitive impairment and Alzheimer’s disease

**DOI:** 10.1038/s41598-024-67177-5

**Published:** 2024-07-29

**Authors:** Nuanyi Liang, Kwangsik Nho, John W. Newman, Matthias Arnold, Kevin Huynh, Peter J. Meikle, Kamil Borkowski, Rima Kaddurah-Daouk, the Alzheimer’s Disease Metabolomics Consortium, Alexandra Kueider-Paisley, P. Murali Doraiswamy, Colette Blach, Arthur Moseley, Siamak Mahmoudiandehkhordi, Kathleen Welsh-Balmer, Brenda Plassman, Andrew Saykin, Shannon Risacher, Gabi Kastenmüller, Xianlin Han, Rebecca Baillie, Rob Knight, Pieter Dorrestein, James Brewer, Emeran Mayer, Jennifer Labus, Pierre Baldi, Arpana Gupta, Oliver Fiehn, Dinesh Barupal, Peter Meikle, Sarkis Mazmanian, Dan Rader, Leslie Shaw, Cornelia van Duijin, Najaf Amin, Alejo Nevado-Holgado, David Bennett, Ranga Krishnan, Ali Keshavarzian, Robin Vogt, Arfan Ikram, Thomas Hankemeier, Ines Thiele, Cory Funk, Priyanka Baloni, Wei Jia, David Wishart, Roberta Brinton, Lindsay Farrer, Rhoda Au, Wendy Qiu, Peter Würtz, Therese Koal, Anna Greenwood, Jan Krumsiek, Karsten Suhre, John Newman, Ivan Hernandez, Tatania Foroud, Frank Sacks

**Affiliations:** 1 West Coast Metabolomics Center, Genome Center, University of California-Davis, Davis, CA 95616 USA; 2 Department of Radiology and Imaging Sciences and the Indiana Alzheimer Disease Center, Indiana University School of Medicine, Indianapolis, IN 46202 USA; 3 Department of Nutrition, University of California-Davis, Davis, CA 95616 USA; 4 Western Human Nutrition Research Center, United States Department of Agriculture-Agriculture Research Service, Davis, CA 95616 USA; 5 Department of Psychiatry and Behavioral Sciences, Duke University, Durham, NC 27708 USA; 6 Institute of Computational Biology, Helmholtz Zentrum München, German Research Center for Environmental Health, Neuherberg, Germany; 7 Baker Heart and Diabetes Institute, Melbourne, VIC 3004 Australia; 8 Baker Department of Cardiovascular Research, Translation and Implementation, La Trobe University, Bundoora, VIC 3086 Australia; 9 Duke Institute of Brain Sciences, Duke University, Durham, NC USA; 10 Department of Medicine, Duke University, Durham, NC USA; 11 Duke University, Durham, USA; 12 Indiana University School of Medicine, Indianapolis, USA; 13 University of Texas Health Science Center San Antonio, San Antonio, USA; 14 Rosa & Co., LLC, San Carlos, USA; 15 University of California-San Diego, San Diego, USA; 16 University of California-Los Angeles, Los Angeles, USA; 17 West Coast Metabolomics Center, University of California, Davis, USA; 18 Icahn School of Medicine at Mount Sinai, New York, USA; 19 California Institute of Technology, Pasadena, USA; 20 University of Pennsylvania, Philadelphia, USA; 21 Oxford University, Oxford, UK; 22 Rush University, Chicago, USA; 23 Erasmus MC, Rotterdam, The Netherlands; 24 Leiden University Metabolomics Center, Leiden, The Netherlands; 25 National University of Ireland, Galway, Ireland; 26 Institute for Systems Biology, Seattle, USA; 27 Purdue University, West Lafayette, USA; 28 University of Hawaii Cancer Center, Honolulu, USA; 29 The Metabolomics Innovation Centre Canada, Edmonton, Canada; 30 University of Arizona, Tucson, USA; 31 Boston University, Boston, USA; 32 Nightingale Health, Helsinki, Finland; 33 Biocrates Life Sciences AG, Innsbruck, Austria; 34 Sage Bionetworks, Seattle, USA; 35 Weill Medical College of Cornell, New York, USA; 36 SUNY Downstate, Brooklyn, USA; 37 National Centralized Repository for Alzheimer’s and Other Dementias, Indianapolis, USA; 38 Harvard School of Public Health, Boston, USA

**Keywords:** Alzheimer’s disease, Mild cognitive impairment, GlycA, Inflammation, Inflammatory biomarker, Metabolomics, Peripheral-central connection, Brain atrophy, Population heterogeneity, Sex differences, Biochemistry, Immunology, Diseases, Medical research, Neurology, Risk factors

## Abstract

Inflammation is an important factor in Alzheimer’s disease (AD). An NMR measurement in plasma, glycoprotein acetyls (GlycA), captures the overall level of protein production and glycosylation implicated in systemic inflammation. With its additional advantage of reducing biological variability, GlycA might be useful in monitoring the relationship between peripheral inflammation and brain changes relevant to AD. However, the associations between GlycA and these brain changes have not been fully evaluated. Here, we performed Spearman’s correlation analyses to evaluate these associations cross-sectionally and determined whether GlycA can inform AD-relevant longitudinal measurements among participants in the Alzheimer’s Disease Neuroimaging Initiative (n = 1506), with additional linear models and stratification analyses to evaluate the influences of sex or diagnosis status and confirm findings from Spearman’s correlation analyses. We found that GlycA was elevated in AD patients compared to cognitively normal participants. GlycA correlated negatively with multiple concurrent regional brain volumes in females diagnosed with late mild cognitive impairment (LMCI) or AD. Baseline GlycA level was associated with executive function decline at 3–9 year follow-up in participants diagnosed with LMCI at baseline, with similar but not identical trends observed in the future decline of memory and entorhinal cortex volume. Results here indicated that GlycA is an inflammatory biomarker relevant to AD pathogenesis and that the stage of LMCI might be relevant to inflammation-related intervention.

## Introduction

Mounting evidence suggests that inflammation and immune dysregulation play a critical role in Alzheimer’s disease (AD) pathogenesis^1^. Inflammation-associated diseases such as metabolic syndromes, diabetes, and cardiovascular diseases are linked to an increased risk of AD^2–6^. In addition, disturbances of circulating markers of inflammation, such as cytokines, endocannabinoids, and oxylipins, have been linked to AD and cognitive decline^7–10^. In pre-clinical models, the peripheral inflammation stimuli, such as a high-fat diet and chronic lipopolysaccharide administration, can lead to AD-related dysregulations in the central nervous system (CNS), including altered blood–brain barrier functionality, changed cerebrovascular properties, neuroinflammation, and amyloid beta (Aβ) pathologies^11–16^. However, complexity does exist, as factors such as sex differences^17,18^ and health status^19,20^ may alter the underlying metabolic connections between peripheral inflammation and brain pathologies. In particular, males and females are susceptible to different inflammatory risk factors throughout their lifetime^21^. The relationship between peripheral inflammation and brain pathologies between males and females may differ over time or throughout the progression of diseases. Generally speaking, despite the observed linkage of peripheral metabolism to central pathologies^13,22–24^, we still need a better understanding of the crosstalk between peripheral inflammation and AD characteristics in the CNS to guide the improvement of inflammatory-related modifiable actions for reducing AD risks and mitigating its pathological trajectories.

Glycoprotein acetyls (GlycA), a biomarker for systemic inflammation, is informative for the level of vascular aging (e.g., due to the significant correlation between GlycA and an indicator of arterial stiffness, aortic pulse wave velocity^25^), the presence of cardiovascular risks (e.g., due to the significant correlation between GlycA and future risk of hypertension and metabolic syndrome^26^, cardiovascular mortality^27^ and other cardiovascular risk markers^28^) and the risk of all-cause mortality^29^. This proton nuclear magnetic resonance (NMR)-based measurement detects the production and further glycosylation of several acute-phase proteins, including alpha-1-acid glycoprotein, alpha-1-antitrypsin, alpha-1-antichymotrypsin, haptoglobin and transferrin^30^. As such, GlycA level captures the overall burden of both the acute and chronic phases of inflammation^28,30^ and has been shown to be informative in the context of inflammatory disorders, such as rheumatoid arthritis^31,32^, lupus^33,34^, psoriasis^35^, atherosclerotic cardiovascular diseases^27,28^, metabolic syndrome^26,36^, type 2 diabetes^37,38^, and inflammatory bowel disease^39^. Compared to the established inflammatory biomarker C-reactive protein (CRP), GlycA may have the advantage of reduced biological variability, as a previous study has shown that the weekly intraindividual and interindividual variations of 23 healthy participants over 5 weeks were substantially lower for GlycA (4.3% and 15.3%, respectively) than for CRP (29.2% and 133.9%, respectively)^30^. In addition, it was shown that compared to CRP, the detection of GlycA was more sensitive among young healthy individuals, less indicative of acute inflammation but more representative of chronic inflammation^26,40^. Meanwhile, the inverse relations between cognitive functions and the level of plasma GlycA^41,42^ or its increases^43^ over years have been reported in studies of younger adults, but such studies are not entirely informative for neurodegenerative diseases that are usually associated with older ages. Therefore, the relationship between GlycA and AD-related biomarkers in older participants has yet to be established.

To further address these knowledge gaps, we aimed in this study to determine the relationship between peripheral inflammation and AD-related biomarkers. We evaluated: (1) differences in GlycA levels in participants at different diagnosis stages along the AD continuum (“Inflammation level indicated by GlycA was elevated along the AD trajectory”); (2) the correlations between baseline GlycA level and executive function (“Baseline GlycA level was correlated with a future decrease in executive function in participants diagnosed with LMCI at baseline”), memory (“Baseline GlycA level was correlated with a future decrease in memory in participants diagnosed with LMCI at baseline”), brain regional brain volumes (“Baseline GlycA level was correlated with a future decrease in entorhinal cortex volume in participants diagnosed with LMCI at baseline” and “Baseline GlycA correlated with baseline brain structural atrophies specifically in female participants diagnosed with LMCI and AD”) and cerebral spinal fluid (CSF) amyloid/tau/neurodegeneration (A/T/N) biomarkers (“Association of plasma GlycA with CSF A/T/N biomarkers”) measured at baseline and measured longitudinally in the follow-up years while considering the influence of diagnosis status and sex on those correlations. To do so, we utilized the data from the Alzheimer’s Disease Neuroimaging Initiative (ADNI) cohort, including baseline NMR measurement of GlycA, baseline and longitudinal executive function composite score, memory composite scores, magnetic resonance imaging (MRI) of brain morphologies and A/T/N biomarkers to perform correlation analysis.

## Methods

### ADNI study participants and data accessibility

Baseline demographic, clinical, cognition, MRI imaging, genetic, biomarkers, cytokines, and multi-omics data (including peripheral GlycA measurement) of the study participants used in the current study were downloaded from the ADNI database (www.adni-info.org), managed through the Laboratory of Neuro Imaging Image & Data Archive (http://adni.loni.usc.edu/). The protocol procedures were approved by the Institutional Review Boards or Research Ethics Boards of the participating institutions. Participants provided signed written informed consent and permission for data access and analysis. All procedures were performed according to relevant guidelines and regulations. The ADNI cohort, launched in 2003 as a public–private partnership and led by Principal Investigator Michael W. Weiner, MD, tests the primary goal of combining serial MRI and positron emission tomography (PET) imaging, biological markers, and clinical and neuropsychological evaluation to measure the progression of mild cognitive impairment (MCI) and early AD.

The diagnosis status of the adult participants included cognitively normal (CN), significant memory concern (SMC), early mild cognitive impairment (EMCI), late mild cognitive impairment (LMCI), and AD. The detailed inclusion and exclusion criteria can be accessed at the ADNI documentation website (https://adni.loni.usc.edu/methods/documents/). In brief, CN participants had Mini-Mental State Exam (MMSE) scores of 24–30 (inclusive) and a Clinical Dementia Rating scale (CDR) score of 0 without any memory complaints. Participants with SMC had self-reported subjective memory concerns but MMSE and CDR scores rated as CN (https://classic.clinicaltrials.gov/ct2/show/NCT01231971). Participants with MCI had an MMSE score of 24–30 (inclusive), a CDR score of 0.5, and a memory complaint, yet did not have sufficient levels of cognition and functional performance impairment to be diagnosed with dementia. Among participants with MCI, EMCI and LMCI were differentiated by different levels of abnormal memory functions, which was indicated by the education-adjusted Wechsler Memory Scale-Revised Logical Memory II subscale, with the cut-off value for LMCI being (a) ≤ 8 for 16 or more years of education, (b) ≤ 4 for 8–15 years of education and (c) ≤ 2 for 0–7 years of education. Participants with AD had an MMSE score of 20–26 (inclusive), a CRD of 0.5 or 1.0, a memory complaint, and their cognition and functional performances met the National Institute of Neurological and Communicative Disorders and Stroke/the Alzheimer’s Disease and Related Disorders Association criteria for probable AD. Exclusion criteria included—but were not limited to—major depression and other significant neurological diseases. Other detailed exclusion criteria can be found on the above-mentioned ADNI study documents website. The dataset contained complete info on GlycA, APOE genotypes, sex, body mass index (BMI), age and education (n = 1506), which was detailed in Table S1. Of the participants, 684 (45.4%) were female; 700 (46.5%) were APOE4 carriers, and 153 (10.2%) were APOE4/4 carriers. In addition, 361 (24.0%) were CN participants, 96 (6.4%) had SMC, 280 (18.6%) had EMCI, 481 (31.9%) had LMCI, and 288 (19.1%) had AD. The age at screening (average ± standard deviation) was 73.1 ± 7.1, baseline BMI was 26.9 ± 4.9, and years of education was 15.9 ± 2.9. The detailed protocols for blood and CSF sample collection and storage can be found on the abovementioned ADNI study documents website.

### Peripheral GlycA measurement

Peripheral GlycA was measured via the Nightingale Health platform using established protocols^44–46^. In brief, serum samples were stored at − 80 °C and thawed overnight at + 4 °C prior to analysis. The samples were then gently mixed, centrifuged, and mixed with an equal amount of an NMR measurement buffer of 75 mmol/L disodium phosphate in 8:2 H_2_O/D_2_O (pH 7.4) with 0.08% sodium 3-(trimethylsilyl)propionate-2,2,3,3-d_4_ and 0.04% sodium azide^44^. The prepared samples were then measured using a Bruker AVANCE III HD 500 MHz spectrometer coupled with a SampleJet cooled robotic sample changer and CryoProbe Prodigy TCI, a cryogenically cooled triple resonance probe head^45^. Quantification results were then obtained from the NMR spectral data via an advanced proprietary software of the Nightingale Health platform^46^.

In addition, the quantitative result of GlycA was log2-transformed and adjusted with medication data prior to further analyses using an Akaike information criterion (AIC) backward stepwise regression method^23,47^. The full list of medication classes and the AIC-selected medications were listed in Table S2. Other confounders, such as age, BMI, sex, education or APOE4 were adjusted as specified in the results.

### Analysis of CRP in plasma and cerebral spinal fluid

Cytokines, including CRP in plasma and CSF were measured using fluorescence-labeled microspheres via a multiplex proteomics method based on the Luminex xMAP platform, with an established threshold of detection and a dynamic range for each analyte^48,49^. The detailed sample preparation was provided by the previous publication, which included steps of sample thawing, the introduction of capture microspheres, the addition of multiplexed mixtures of reporter antibodies (biotinylated), multiplex development via the streptavidin–phycoerythrin solution, and final volume adjustment^49^. Prior to statistical analysis, cytokines were normalized and medication adjusted as above.

### MRI brain imaging data acquisition

MRI protocol and data were accessed through the ADNI website (https://adni.loni.usc.edu/). In brief, MRI imaging was acquired using a 1.5 T or 3 T MRI scanner with the sequences of T1 and dual echo T2-weighted imaging (ADNI 1), or a 3 T MRI scanner with fully sampled and accelerated T1-weighted imaging in addition to 2D FLAIR and T2*-weighted imaging (ADNI GO/2). To process MRI scans and extract whole brain and ROI (region of interest)-based neuroimaging measurements determined by automated segmentation and parcellation including volumes and cortical thickness, FreeSurfer V6, a widely employed automated MRI analysis approach, was used^50^. After the cortical surface was reconstructed, the cortical thickness at each vertex was calculated by taking the Euclidean distance between the grey/white boundary and the grey/cerebrospinal fluid (CSF) boundary at each vertex on the surface.

### Cognitive function and memory measurement

A composite score for measuring overall executive functioning (EF) was previously developed and validated^51^. In brief, the composite score was constructed via an iterative process that included the confirmatory factor analysis to build a model and the reviewing of findings to construct a revised model. The model was built using ADNI baseline data, based on the Category fluency (animals) test, Category fluency (vegetables) test, Trails A and Trails B test, Digit span backwards test, Wechsler Adult Intelligence Scale—Revised Digit Symbol Substitution test, and the five Clock Drawing items test (circle, symbol, numbers, hands, and time).

A composite score for measuring the overall memory (MEM) was previously developed and validated^52^. In brief, MEM was derived from two versions of the longitudinal Rey Auditory Verbal Learning Test, three versions of the AD Assessment Schedule—Cognition, the MMSE, and Logical Memory data, using a single factor model.

### Amyloid/Tau/Neurodegeneration (A/T/N) biomarker measurement in CSF

A/T/N biomarkers, Aβ_1-42_, t-tau, and p-tau_181p_ in CSF samples were measured via a fully automated Roche Elecsys immunoassay, in which a sample preparation procedure with two incubation steps was used^53,54^.

### Statistical analyses

All statistical analyses were performed in JMP® Pro 16 and 17 (SAS Institute Inc., Cary, NC). We used the standard least-square method for analysis of covariance (ANCOVA) or calculating the residual of variable controlling confounders. ANCOVA was applied to compare the differences in means of GlycA and CRP in both plasma and CSF, followed by Tukey HSD post hoc analysis to evaluate the significant differences among various groups. The effect size of the comparison between any two groups was evaluated using Cohen’s term (d_Cohen_), which was calculated by the differences in means divided by the standard deviation of all measurements in both groups.

To investigate non-linear associations, Spearman’s correlations were performed on residuals of specific measurements while controlling confounders, namely APOE4, age, BMI, education level, sex, diagnosis status and/or medications. For the MRI measurement of brain volumes, we adjusted for the confounders of magnet types of MRI and intracranial volume (log2-transformed). A linear model with sex or diagnosis status interaction with GlycA was additionally applied to test whether the observed associations were different between sexes or among diagnosis statuses.

Additional Spearman’s correlation analyses were performed to evaluate the relationship of baseline GlycA with cognitive functions and brain regional volumes in the future follow-up years with the same confounders adjusted as above, in addition to adjusting to the baseline value of these measurements. The analysis was performed with stratification of diagnosis status at baseline because participants with differing diagnosis status at baseline experienced different trajectories of brain structural and cognitive changes in the follow-up years^55^, which might result in differential relationships between GlycA and these brain measurements. For multiple comparisons, the false discovery rate (FDR) correction using the Benjamini and Hochberg method (q = 0.2) was applied^56^.

### Ethics approval and consent to participate

The protocol procedures were approved by the Institutional Review Boards or Research Ethics Boards of the participating institutions. Participants provided signed written informed consent and permission for data access and analysis.

## Results

### Inflammation level indicated by GlycA was elevated along the AD trajectory

**Figure 1:**
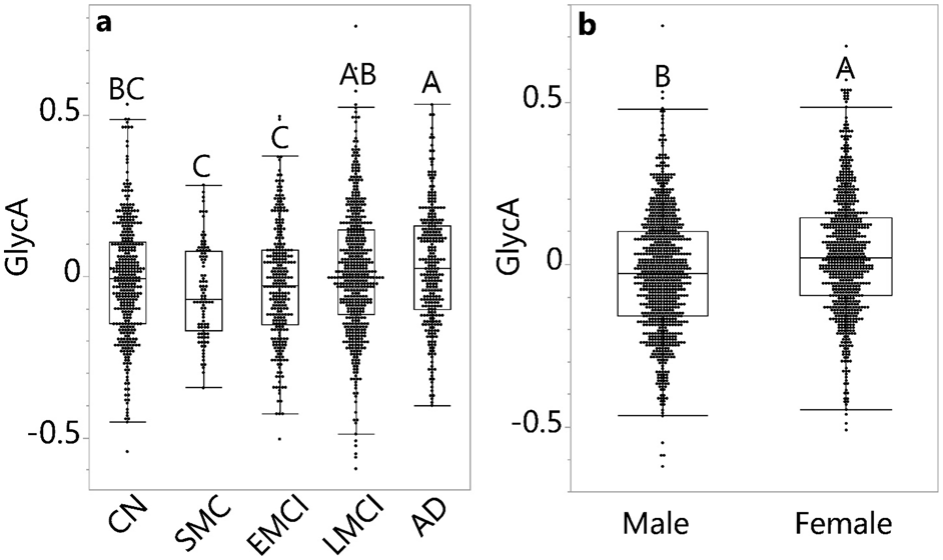
GlycA levels in ADNI participants. GlycA level in (**a**) groups of various diagnosis statuses, adjusted for sex and (**b**) sexes, adjusted by diagnosis status. (**a**) GlycA level was log2-transformed and adjusted for medications, sex, age, APOE4, BMI, and education level. (**b**) GlycA level was log2-transformed and adjusted for medications, diagnosis status, age, APOE4, BMI, and education level. ANCOVA and Tukey HSD post hoc analysis were performed; levels not labeled with the same letter differed significantly (*p* < 0.05) (e.g., the groups in the same figure labeled with A and B were significantly different from each other, but the ones labeled with A and AB were not significantly different). Values in the bar graph were shown as the mean residual GlycA level ± standard error. There was no sex-diagnosis status interaction in a full factorial model. AD: Alzheimer’s disease; ANCOVA: Analysis of covariance; BMI: Body mass index; CN: Cognitively normal; EMCI: Early mild cognitive impairment; GlycA: Glycoprotein acetyls; HSD: Honestly significant difference; LMCI: Late mild cognitive impairment; SMC: Significant memory concerns.

To evaluate the level of peripheral inflammation along AD progression, we applied an ANCOVA model that tested GlycA level against diagnosis groups (CN, SMC, EMCI, LMCI and AD) and controlled for the presence of APOE4 allele, age, BMI, education and sex as covariates (Fig. 1a). The sex-diagnosis group interaction was additionally applied to test whether sex influences the pattern of GlycA levels along the disease progression and it was not significant (*p* = 0.579), and thus the interaction term was removed from further analysis.

GlycA level increased in the participants at more severe disease stages (diagnosis group *p* < 0.001). In particular, participants with AD had higher GlycA levels compared to CN participants and participants with SMC and EMCI. Participants with LMCI had higher GlycA levels than did participants with SMC and EMCI. In the same model, GlycA was positively associated with BMI (*p* < 0.001, β_estimate_ = 0.01) and negatively associated with education (*p* < 0.001, β_estimate_ = − 0.01). GlycA was also higher in females than in males (*p* < 0.001, d_Cohen_ = 0.3, Fig. 1b). GlycA was not influenced by the presence of APOE4 (*p* = 0.227) or age (*p* = 0.143).

Additionally, we evaluated the relationship between plasma GlycA and plasma CRP using Spearman’s rank order correlation to compare the NMR-derived inflammation indicator to the commonly used peripheral inflammation marker (Fig. S1A). Moreover, we correlated plasma GlycA and CRP in CSF to evaluate the association between peripheral and central inflammation (Fig. S1B). GlycA was positively associated with CRP in both plasma (*p* < 0.001, ρ = 0.445) and CSF (*p* < 0.001, ρ = 0.370). In contrast, CRP in both plasma and CSF did not differ among the different diagnosis groups (*p* = 0.192 and *p* = 0.832), and only CRP in plasma was higher in females compared to males (*p* = 0.015, d_Cohen_ = 0.2) (Fig. S2), where fewer participants had their CRP measured (n for each group: in blood: n = 50 (CN), 0 (SMC), 0 (EMCI), 337 (LMCI), and 97 (AD); in CSF: n = 74 (CN), 0 (SMC), 0 (EMCI), 133 (LMCI), and 60 (AD)). The lack of significant differences in plasma CRP among diagnosis groups due to reduced data coverage was supported by the result that when adjusting plasma CRP, GlycA levels were no longer significantly different among the various diagnosis groups in the abovementioned ANCOVA model (*p* = 0.126).

### Baseline GlycA level was correlated with a future decrease in executive function in participants diagnosed with LMCI at baseline

To interrogate whether baseline GlycA can predict future cognitive decline, we evaluated the correlation of baseline GlycA with the longitudinal composite scores for EF^51^. The relationship between baseline GlycA and EF in follow-up years was evaluated using Spearman’s rank order correlation analyses, adjusting for baseline EF, sex, age, APOE4 genotype, BMI, education and follow-up years, and treating participant ID as a random factor. In addition, due to the differential connections observed between central and peripheral changes in different stages of AD progression^57^, the analyses were performed with stratification of diagnosis status across participants of CN, SMC, EMCI, LMCI, and AD (Table S3). Linear mixed models were used to confirm these analyses (Tables S4 and S5).

**Figure 2:**
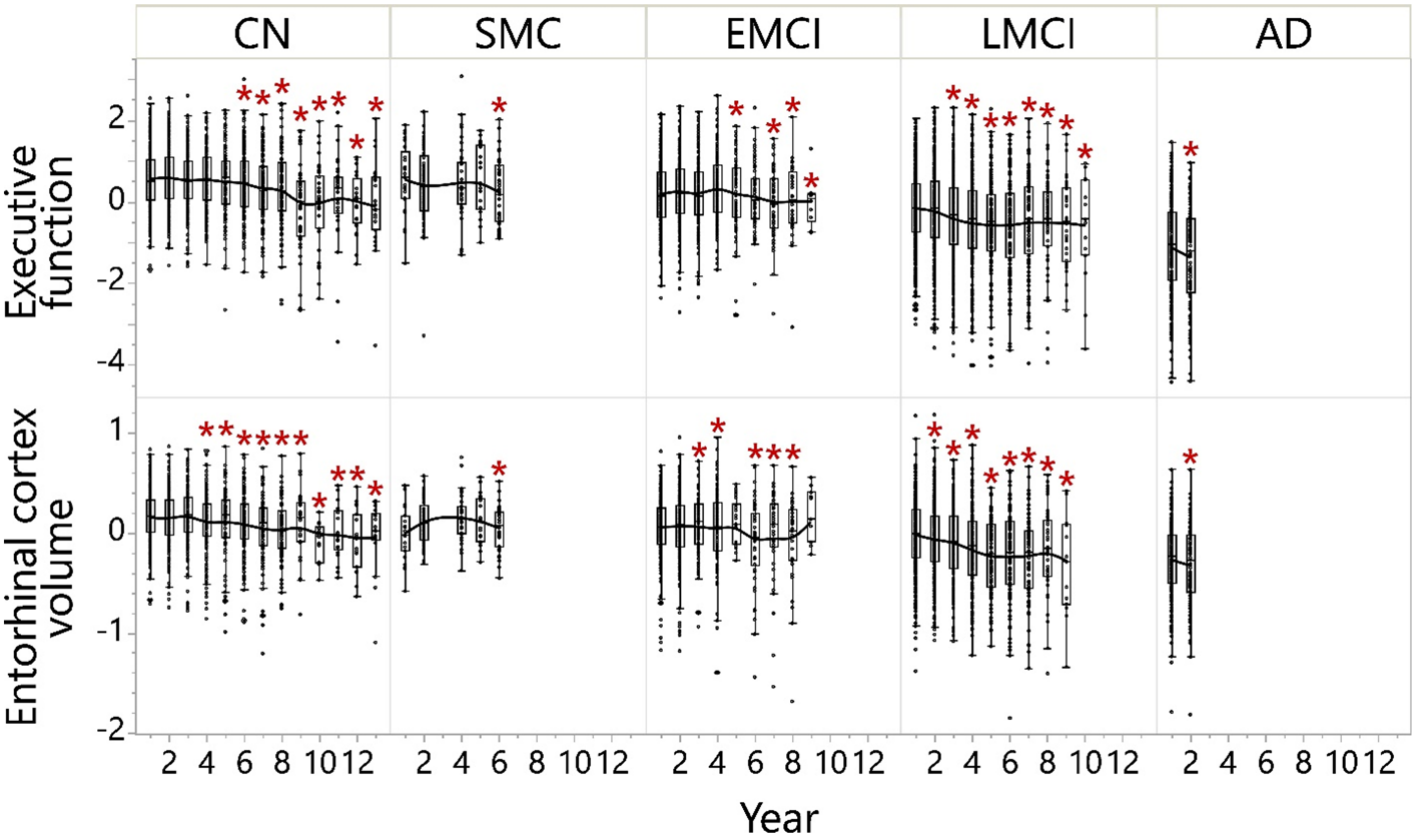
Executive function and entorhinal cortex volume decline in ADNI participants. Participants diagnosed with LMCI at baseline started to have the most continuous executive functional decline on and after 3-year follow-up and continuous entorhinal cortex volume decline on and after 2-year follow-up, which was well distinguished from CN participants and participants with SMC and EMCI. Executive function was executive function score adjusted for sex, APOE4, education, BMI at visit and age at screening. The ANCOVA results comparing different time points within each diagnosis group are shown in the heatmap of Fig. S3; the ANCOVA results comparing EF of different diagnoses at baseline are shown in Fig. S4. Entorhinal cortex volume was entorhinal cortex volume adjusted for intracranial volume (log2), magnet type, sex, APOE4, education, BMI at visit and age at screening. The ANCOVA results comparing different time points within each diagnosis group are shown in the heatmap of Fig. S7; the ANCOVA results comparing the entorhinal cortex volume of different diagnoses at baseline are shown in Fig. 5. Results annotated with * were at a significantly different level compared to Year 1 within the diagnosis group (Figs. S3 and S7). AD: Alzheimer’s disease; ANCOVA: Analysis of covariance; BMI: Body mass index; CN: Cognitively normal; EMCI: Early mild cognitive impairment; LMCI: Late mild cognitive impairment; SMC: Significant memory concerns.

**Figure 3:**
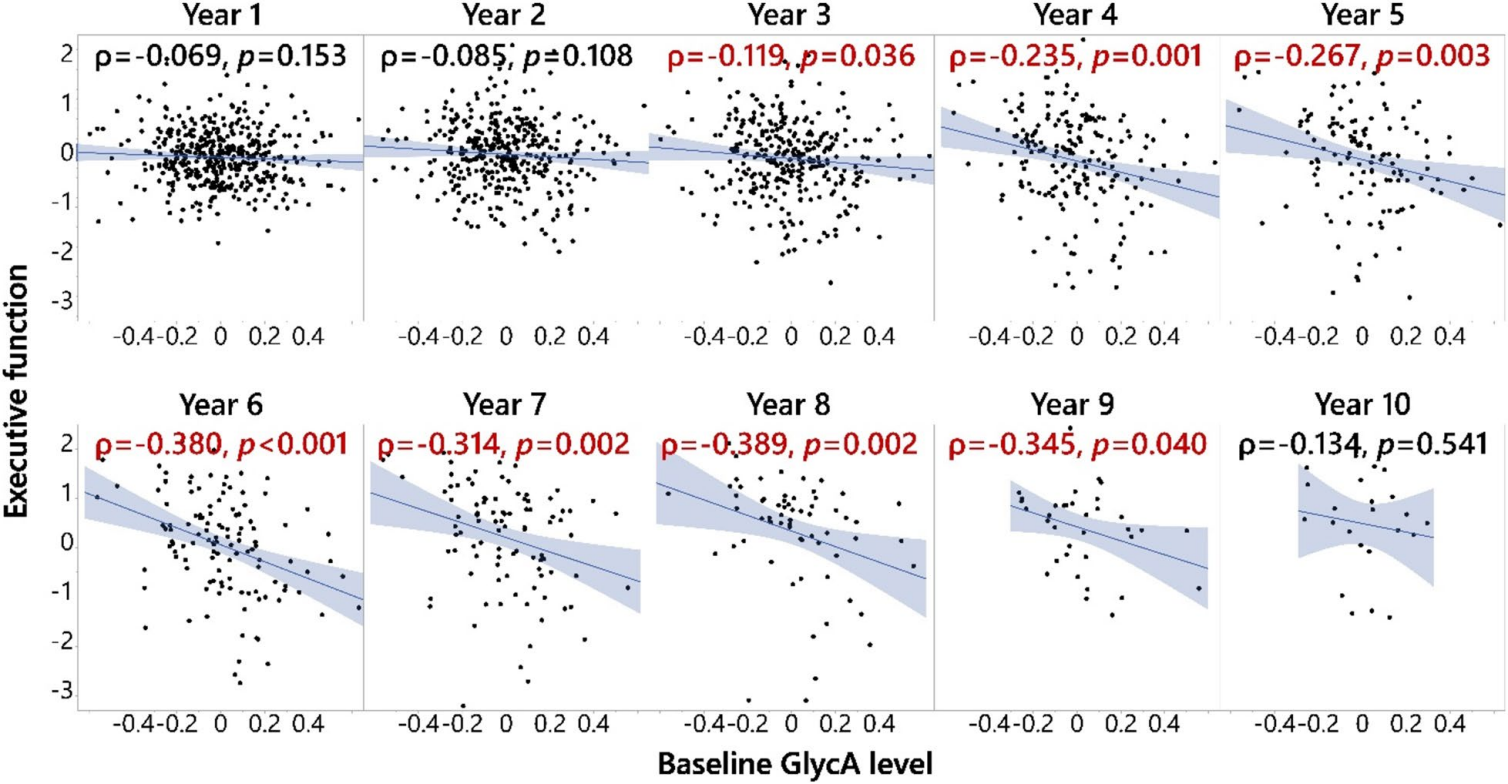
Baseline GlycA level associated with future executive function declines in LMCI patients. Baseline GlycA level was associated with executive function composite score (EF) declines in late mild cognitive impairment (LMCI) patients at 3–9 years of follow-up. Spearman’s rank order correlation was performed on residuals of EF and GlycA, and the analyses were stratified by diagnosis status. EF at different years were adjusted for baseline EF level, screening age, follow-up year, BMI at the time, APOE4, sex, and education, treating participant ID as random factors; baseline GlycA level was log2-transformed and adjusted for medication, screening age, baseline BMI, APOE4, sex, and education. All significant results shown in the figure passed FDR correction using the Benjamini and Hochberg method (q = 0.2)^56^. The analysis was performed on participants with different diagnosis statuses (Table S3), but only in the participants with LMCI was EF at continuous follow-up years negatively associated with baseline GlycA level and thus shown here. In addition, the LMCI-GlycA interaction was confirmed using a linear mixed model (*p* = 0.025, Table S4). Therefore, the analysis results for the participants with LMCI are shown here. A full factorial linear mixed model was used to show that there were no sex-GlycA interactions controlling DX (*p* = 0.864). BMI: Body mass index; DX: Diagnosis at baseline; GlycA: Glycoprotein acetyls; ID: Identification.

ADNI data with the baseline GlycA measurement enabled us to probe cognitive changes for up to a 13-year follow-up (Fig. 2 and Fig. S3). Participants with differing diagnosis status at baseline experienced different trajectories of brain structural and cognitive changes in the follow-up years (Fig. 2). In particular, participants diagnosed with LMCI at baseline experienced the earliest EF declines (defined as the longitudinal EF significantly deviated from Year 1 follow-up) in follow-up years among participants who were not diagnosed with AD at baseline, starting on Year 3 (Fig. 2 and Fig. S3). Therefore, we specifically investigated the correlations between baseline GlycA level and EF in the different years of follow-up among participants diagnosed with LMCI at baseline, while other diagnosis statuses have also been explored (Table S3). The LMCI-GlycA interaction to predict EF was confirmed using a linear mixed model (*p* = 0.025, Table S4). In participants with LMCI, baseline GlycA was negatively associated with EF in years 3–9 of follow-up (Fig. 3, with year 3 (*p* = 0.036, ρ = -0.119), year 4 (*p* = 0.001, ρ = − 0.235), year 5 (*p* = 0.003, ρ = − 0.267), year 6 (*p* < 0.001, ρ = − 0.380), year 7 (*p* = 0.002, ρ = − 0.314), year 8 (*p* = 0.002, ρ = − 0.389) and year 9 (*p* = 0.040, ρ = − 0.345)). Additionally, GlycA-EF association among participants with LMCI was not sex-dependent (*p*_interaction_ = 0.833). The negative correlation between longitudinal EF and baseline GlycA in participants diagnosed with LMCI at baseline was confirmed using a linear mixed model (Table S5).

Of note, only a weak association (*p* = 0.049, ρ = − 0.172) was observed between the baseline GlycA and baseline EF in female participants with AD (Fig. S4). No associations were detected in females in other diagnostic groups, or in males. Therefore, the lack of sex effects on the GlycA-EF relationship was consistent between cross-sectional and longitudinal observations.

### Baseline GlycA level was correlated with a future decrease in memory in participants diagnosed with LMCI at baseline

To investigate whether memory function follows a pattern similar to executive function, similar analyses to the above were performed on baseline GlycA and longitudinal composite scores for MEM across participants of CN, SMC, EMCI, LMCI, and AD (Table S3)^52^. Participants diagnosed with LMCI at baseline experienced the earliest continuous cognitive declines (i.e., cognition significantly deviated from Year 1 follow-up) in follow-up years among participants who were not diagnosed with AD at baseline, starting in Year 2 (Fig. S5). In these participants, baseline GlycA was negatively associated with MEM in years 5–8 of follow-up (Fig. S6, with year 5 (*p* = 0.036, ρ = − 0.183), year 6 (*p* = 0.037, ρ = − 0.186), year 7 (*p* = 0.019, ρ = − 0.235) and year 8 (*p* = 0.036, ρ = − 0.262)). However, these associations did not pass FDR correction and were weaker compared to the associations with EF, which was confirmed using a linear mixed model (Table S5). There was also no significant LMCI-GlycA interaction to predict MEM, which was confirmed using a linear mix model (*p* = 0.441, Table S4). Additionally, GlycA-MEM association among participants with LMCI was not sex-dependent (*p*_interaction_ = 0.471). Unlike baseline EF, there was no association between baseline GlycA and baseline MEM in either sex or in any diagnosis group (*p* > 0.05).

### Baseline GlycA level was correlated with a future decrease in entorhinal cortex volume in participants diagnosed with LMCI at baseline

**Figure 4:**
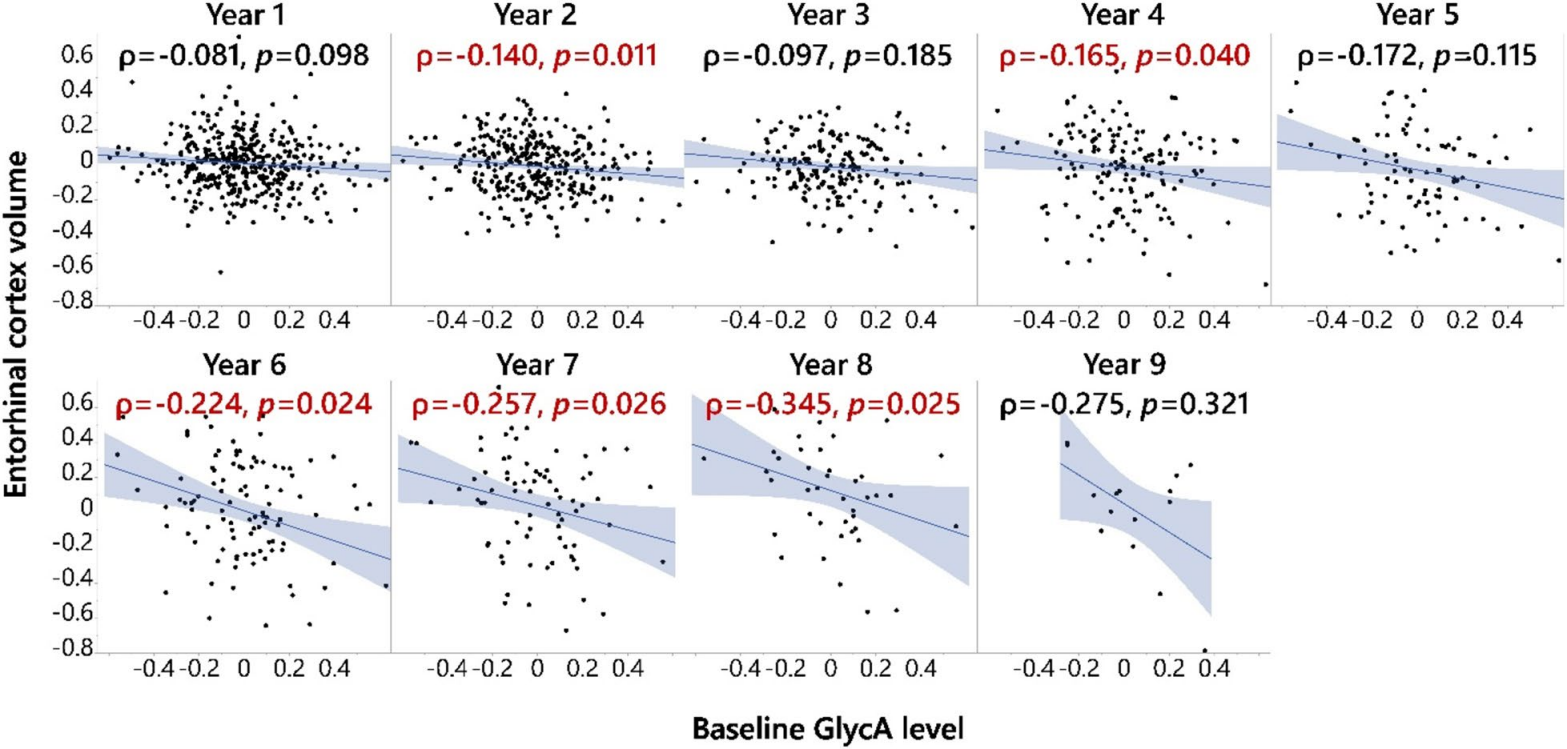
Baseline GlycA level associated with future entorhinal cortex volume declines in LMCI patients. Baseline GlycA level was associated with entorhinal cortex volume declines in late mild cognitive impairment (LMCI) patients at the 2nd, 4th, and 6–8th years of follow-up. Spearman’s rank order correlation was performed on residuals of entorhinal cortex volume and GlycA, and the analyses were stratified by diagnosis status. Entorhinal cortex volume at different years was adjusted for baseline entorhinal cortex volume, screening age, follow-up year, BMI at the time, APOE4, sex, and education, treating participant ID as random factors (all MRI volumes were log2-transformed and adjusted for intracranial volume and magnet type); baseline GlycA level was log2-transformed and adjusted for medication, screening age, baseline BMI, APOE4, sex, and education. All significant results shown in the figure passed FDR correction using the Benjamini and Hochberg method (q = 0.2)^56^. The analysis was performed on participants with different diagnosis statuses (Table S6), but only in the participants with LMCI was entorhinal cortex volume in three continuous follow-up years negatively associated with baseline GlycA level. Therefore, the analysis results for the participants with LMCI are shown here. In addition, the LMCI-GlycA interaction was confirmed with a linear mixed model (*p* = 0.005, Table S4). A similar full factorial linear mixed model was used to show that there were no sex-GlycA interactions controlling DX (*p* = 0.564). BMI: Body mass index; DX: Diagnosis at baseline; GlycA: Glycoprotein acetyls; ID: Identification; MRI: Magnetic resonance imaging.

To identify the structural basis of the MEM and EF patterns observed above, we performed a similar analysis on MRI-measured brain regional volumes with additional adjustment for MRI methods and intracranial volume, across participants of CN, SMC, EMCI, LMCI, and AD (Table S6). We therefore investigated whether baseline GlycA can predict the further decline in brain regional volumes using the above-described approach. Participants diagnosed with LMCI at baseline experienced the earliest continuous memory declines (i.e., memory significantly deviated from Year 1 follow-up) in follow-up years among participants who were not diagnosed with AD at baseline, starting in Year 2 (Fig. 2 and Fig. S7). Negative associations between baseline GlycA and entorhinal cortex volume was observed for the follow-up years 2 (*p* = 0.011, ρ = − 0.140), 4 (*p* = 0.040, ρ = − 0.165), 6 (*p* = 0.024, ρ = − 0.224), 7 (*p* = 0.026, ρ = − 0.257) and 8 (*p* = 0.025, ρ = − 0.345) (Fig. 4). The negative correlation between longitudinal entorhinal cortex volume and baseline GlycA in participants diagnosed with LMCI at baseline was confirmed using a linear mixed model (Table S5). The LMCI-GlycA interaction to predict entorhinal cortex volume was confirmed using a linear mixed model (*p* = 0.005, Table S4). Additionally, GlycA-entorhinal cortex volume association among participants with LMCI was not sex-dependent (*p*_interaction_ = 0.333). Similar associations were not observed with other brain regional volumes (Table S6).

### Baseline GlycA correlated with baseline brain structural atrophies specifically in female participants diagnosed with LMCI and AD

**Figure 5:**
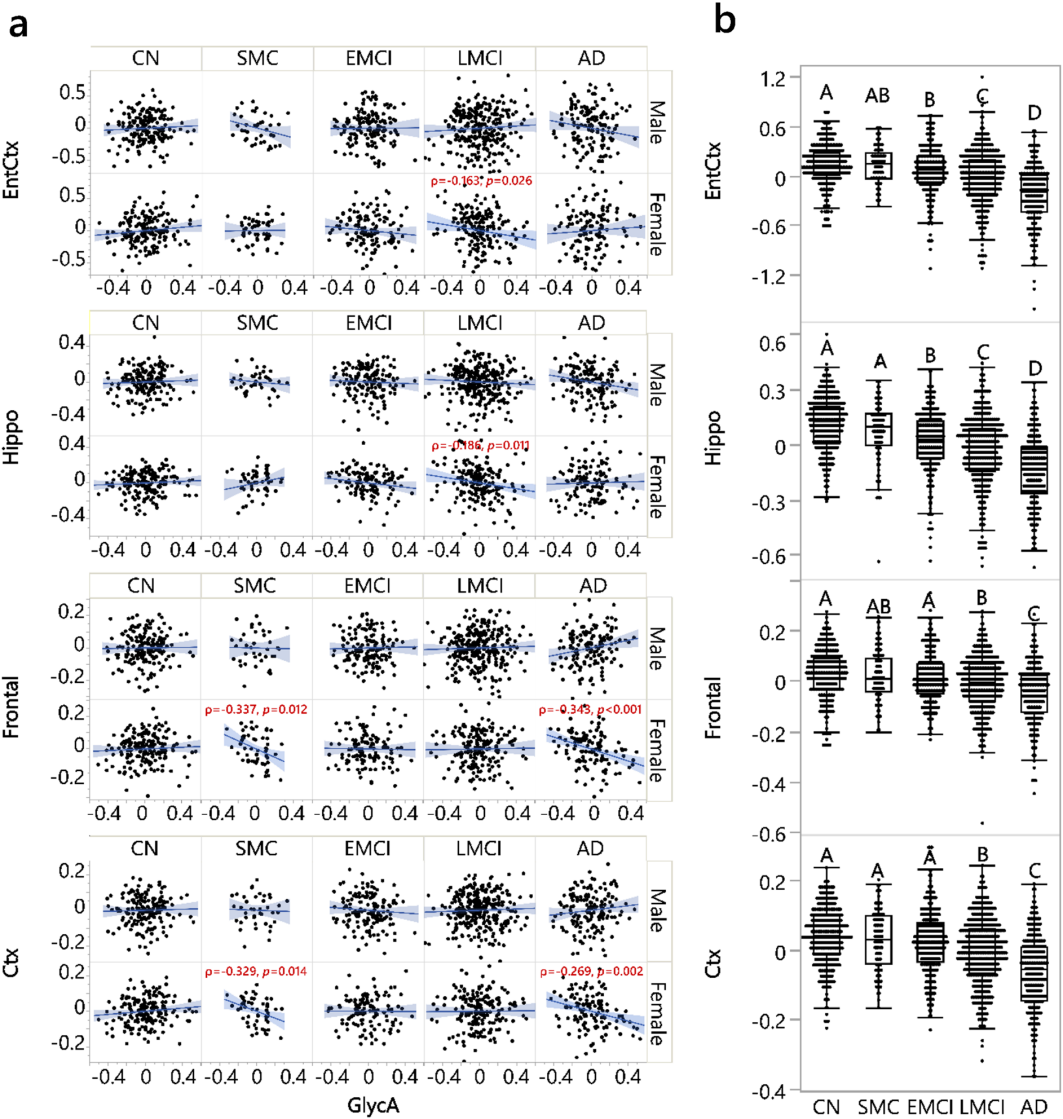
GlycA cross-sectionally associated with regional brain volumes in females with SMC, LMCI and AD. The full results are listed in Tables S7 and S9 and the present figure illustrates selected representative examples. (**a**) Significant associations were found between GlycA and volumetric measurements of brain regions, i.e., entorhinal cortex (EntCtx), hippocampus (Hippo), frontal lobe (Frontal), and cerebral cortex grey matter (Ctx). Spearman’s rank order correlation was performed between the residual of GlycA (log2-transformed, adjusted for medications, APOE4, age, BMI, and education) and residuals of whole brain regional volumes (log2-transformed, adjusted for log2-intracranial volume, magnet strength/scan type, APOE4, age, BMI, education); the analysis was performed stratified by sex and diagnosis groups. All significant results shown in the figure passed FDR correction using the Benjamini and Hochberg method (q = 0.2)^56^. (**b**) This panel indicates different levels of these brain volumetric measurements, adjusting for the same confounders without stratifications (Table S9). There were no significant sex-diagnosis stages interactions (*p*_interaction_ = 0.284 (EntCtx), 0.244 (Hippo), 0.324 (Frontal) and 0.398 (Ctx)) for brain volumetric measurements and therefore this panel is not divided by sex. N: for males: CN: 178, SMC: 40, EMCI: 154, LMCI: 292, and AD: 154; for females: CN: 183, SMC: 55, EMCI: 125, LMCI: 187, and AD: 131. AD: Alzheimer’s disease; ANCOVA: Analysis of covariance; BMI: Body mass index; CN: Cognitively normal; EMCI: Early mild cognitive impairment; GlycA: Glycoprotein acetyls; LMCI: Late mild cognitive impairment; SMC: Significant memory concerns.

To identify the cross-sectional relationship between GlycA and regional brain atrophies measured by MRI, we used Spearman’s rank order correlation with sex and diagnostic group stratification (Table S7, Fig. 5). Bilateral total/mean measurements were used to examine the correlation of brain atrophy with GlycA, with measures in the left and right hemispheres showing similar results (Table S8). In addition, rather than using thickness, we mainly used regional volumes to describe brain atrophy due to its stronger sensitivity^58^, though we did provide both results (Table S8). Comparisons of volumetric measurements among participants of different diagnosis statuses were performed using an ANCOVA model (Table S9).

In females with AD, GlycA was negatively associated with the frontal lobe volume (*p* < 0.001, ρ = − 0.343), cerebral cortex grey matter volume (*p* = 0.002, ρ = − 0.269), global grey matter mean volume (*p* = 0.002, ρ = − 0.267), sensory motor volume (*p* = 0.014, ρ = − 0.214), and parietal lobe volume (*p* = 0.011, ρ = − 0.222) (Table S7). Except for parietal lobe volume, all of these GlycA-brain morphology associations were specific for females with AD, compared to males with AD. The specificity of the association was measured using a linear factorial model with GlycA-sex interaction, stratified by diagnostic group, with results for AD participants including *p*_*interaction*_ < 0.001 for the frontal lobe volume, *p*_*interaction*_ = 0.008 for cerebral cortex grey matter volume, *p*_*interaction*_ = 0.007 for grey matter mean volume, and *p*_*interaction*_ = 0.020 for sensory motor volume (Table S7).

In addition, in females with LMCI, GlycA was negatively associated with hippocampus volume (*p* = 0.011, ρ = − 0.186) and entorhinal cortex volume (*p* = 0.026, ρ = − 0.163). In females with SMC, GlycA was negatively associated with cerebral cortex grey matter volume (*p* = 0.014, ρ = − 0.329), frontal lobe volume (*p* = 0.012, ρ = − 0.337), and mean grey matter volume (*p* = 0.011, ρ = − 0.341). No correlation passing FDR correction was observed in the CN or EMCI diagnostic groups, nor in male participants of any diagnosis status. None of the GlycA-sex interactions in non-AD groups and GlycA-diagnosis interactions in both sexes were significant after FDR correction (Table S7).

It is important to note that brain region atrophy was not uniform along AD disease progression (Fig. 5b, Table S9). While areas such as the entorhinal cortex and hippocampus had significantly decreased volumes compared to CN as early as the EMCI stage (*p* < 0.05), other volumetric measurements—such as those in cerebral cortex grey matter and the frontal lobe—only started to have significantly decreased volumes compared to CN in LMCI. Consistent with this, the correlations between GlycA and hippocampus volume and between GlycA and entorhinal cortex volume were found in an earlier diagnosis stage in females (i.e., LMCI) compared to GlycA correlation with cerebral cortex grey matter volume and frontal lobe volume, which were found in a later stage (i.e., AD). However, such disease state patterns were not inclusive, meaning that not all brain anatomical region changes along disease progression coincided with their correlation with GlycA. An example of this is the early change of medial temporal lobe volume at the EMCI stage without a significant correlation with GlycA, found in participants with any diagnosis status.

### Association of plasma GlycA with CSF A/T/N biomarkers

We investigated the relationship between GlycA in plasma and A/T/N biomarkers in CSF using Spearman’s rank correlation as described above. No association was observed between plasma GlycA and A/T/N biomarkers in CSF, including the biomarkers Aβ 42, pTau/Aβ 42, pTau, total Tau, and total Tau/Aβ 42 (Table S10). GlycA in plasma was also not associated with A/T/N biomarkers in CSF in all participants in the continuous future follow-up years (Table S11).

## Discussion

The current study describes associations between the level of peripheral inflammation and AD-related structural and cognitive changes in the CNS. Importantly, these peripheral-central correlations manifest primarily in the later stages of the progression toward AD, which emphasizes the diagnosis-status-specific heterogeneity in the relationship between peripheral inflammation, cognition, and brain atrophy. Our findings point to peripheral inflammation as a risk factor for future cognitive decline and brain atrophy in both males and females, which opens the door to the identification of a population at risk as well as potential therapeutic interventions relevant to further inflammatory-related cognitive decline.

Abnormal cognition and memory decline are important markers of AD pathogenesis^59^, and the structural and functional degeneration of the entorhinal cortex often indicate the early changes in AD development, which implies the particular vulnerability of this region to AD pathogenesis^60,61^. Accordingly, our results have shown that the peripheral inflammation at baseline correlates with decreasing executive functions, decreasing memory and decreasing entorhinal cortex volume in the follow-up years. The decreased entorhinal cortex volume may provide an inflammatory-related structural basis to explain the observed declines in brain functions that are associated with inflammation.

In addition, such correlations between baseline GlycA and future declines in executive functions, memory and entorhinal cortex volume are restricted to the participants diagnosed with LMCI at baseline. It happened to coincide with the observation that among non-AD participants, the LMCI diagnosis group had the earliest brain function and structural decline in the follow-up years. Mechanistically, peripheral cytokines can directly enter the CNS and/or interact with the blood–brain barrier through multiple pathways^13,62^. Meanwhile, under pathological conditions, peripheral immune cells can migrate across the blood–brain barrier^63–65^ and modulate microglia function^66,67^, a key player in CNS inflammation and AD development. As results, peripheral inflammation stimuli can modulate the expression of inflammation-related genes in the brain^19^ and exacerbate CNS neurodegeneration^53^. Furthermore, brain inflammation has been suggested to have differential effects along the AD progression^20^. For example, in the AD rodent model of APPPS1 mice, the lack of neuroinflammation-related gene TREM2 expression (i.e., TREM2 deficiency) lowered amyloid pathologies at the earlier stage of disease development but worsened it at the later stage. Specifically at the later stage, TREM2 deficiency reduced the proliferation of myeloid cells and the gene transcription levels related to inflammation, such as IL-1β and TNF-α^20^. These studies support the notion that certain inflammation components can be protective at the beginning of the progression toward AD, but detrimental to CNS homeostasis at the later stages^20,68^. Such a notion may explain the relationship between baseline GlycA and AD-related biomarkers in the follow-up years for participants with LMCI. It may also explain the cross-sectional correlation between baseline GlycA and baseline AD-related biomarkers in the female participants with LMCI and AD. This is due to the consideration that the depletion of estrogens after menopause may lead to additional vulnerability to oxidative stress in women^69,70^, and thus that the relationship between oxidative stress and neurodegeneration may differ in older females compared to males^71^.

Sex differences were observed in the cross-sectional correlation between peripheral inflammation and brain structures. This was different from the relationship between baseline peripheral inflammation and brain structures in the follow-up years, in which no sex-dependent effect was observed. While causation cannot be inferred from our observations, such a difference in peripheral-central connections between cross-sectional and longitudinal analysis may be due to the difference between acute and chronic inflammation, both of which can be captured by GlycA^28,30^. Sexual dimorphism in neurological disorders has been associated with differential microglia sub-populations, including disease-associated microglia and activated response microglia^72–74^, as well as the microglial involvement in AD-related pathologies such as neuroinflammation^75^, Aβ accumulation^72^ and tau pathologies^75–77^. In a rodent study, the identical acute peripheral inflammatory stimuli caused a more substantial pro-inflammatory response in the aged brains of females compared to males^18^. However, it is not clear whether the aged brains of males and females respond to the chronic peripheral stimuli in a similar manner^19,78^, considering that males and females overall manifest different fluctuations in peripheral inflammation over a lifetime^21^. Further longitudinal studies on aged models or populations with chronic inflammation are therefore warranted to clarify the potential sex effect of inflammation on AD development.

Additionally, while we observed the correlation between GlycA and brain structure, and between GlycA and cognitive changes, there was no correlation between GlycA and CSF biomarkers for Aβ and tau pathologies. Similar observations on the lack of correlations between peripheral inflammatory indicators and AD pathologies were reported before, which can be accounted for by factors such as the temporal dynamics of these inflammatory cytokines and AD progression (reviewed in^7^). Moreover, additional regulation of peripheral inflammation and AD pathologies must occur in the CNS, which is supported by the evidence found in animal research: for example, the level of amyloid deposition in the brain is negatively associated with CSF Aβ level^79^. However, in an amyloid precursor protein knock-in mouse model, peripheral inflammation increased Aβ deposition in the brain^80^, but interrupted Aβ clearance from CSF to blood^80^. Such an interaction could explain the lack of changes in CSF Aβ under the peripheral inflammation stimuli^80^, consistent with our current observation. In addition, peripheral inflammation affects the transmission of Tau proteins among brain regions^81^, but how these processes collectively regulate the level of Tau pathology in CSF is not clear. Overall, the mechanisms that regulate the complex crosstalk between peripheral inflammation and different AD pathologies warrant further investigation.

It should be pointed out that our analyses were limited to observational findings instead of causative ones. Replications on independent cohorts are required for confirmation. In addition, the comorbidity of AD pathologies and the diagnosis status of inflammatory diseases such as diabetes and metabolic syndrome were not discussed here, considering that GlycA captured the systemic inflammation level^28,30^ regardless of the specific diagnosis statuses of those inflammatory diseases. Nevertheless, the comorbidity of diabetes and AD-related pathologies has been reported in a previous study performed using the ADNI data^82^. Furthermore, some of the stratifications resulted in a relatively small sample size per group (e.g. SMC), but these stratifications remain to be used considering the different risks of dementia in different populations^83,84^. Further analyses are warranted to investigate the extent of this population heterogeneity and the possibility of combining subgroups to achieve higher statistical power. Meanwhile, other factors that can contribute to population heterogeneity, such as race and ethnicity, cannot be investigated well with the current dataset but can be addressed through future opportunities using cohorts involving diverse populations (e.g. ADNI4, which is currently under recruitment). Additionally, other composite scores to measure different cognition domains, such as language composite (LAN) and visuospatial composite scores (VS) are available in ADNI^85^, but the systemic inspection of their relationship with GlycA was outside of this paper’s scope.

## Conclusions

Our study describes connections between peripheral inflammation and cognitive and structural AD-related changes in the brain. Moreover, it highlights the implication of systemic inflammation at earlier time points on the later stage toward AD and highlights the potentially higher sensitivity or susceptibility of females, compared to males, to systemic inflammation-related brain changes. These findings contribute to our understanding of disease heterogeneity and lay the groundwork for more personalized approaches toward disease treatment.

### Supplementary Information

Supplementary Information.

## Data Availability

The datasets supporting the conclusions of this article are available in the ADNI database (www.adni-info.org), managed through the Laboratory of Neuro Imaging Image & Data Archive (http://adni.loni.usc.edu/). The data is available for authorized investigators.

## Abbreviations

Aβ: Amyloid beta peptide
AD: Alzheimer’s disease
ADNI: Alzheimer’s Disease Neuroimaging Initiative
ANCOVA: Analysis of covariance
BMI: Body mass index
CDR: Clinical Dementia Rating scale
CN: Cognitively normal
CNS: Central nervous system
CRP: C-reactive protein
CSF: Cerebral spinal fluid
Ctx: Cerebral cortex grey matter
DX: Diagnosis at baseline
EF: Executive functioning composite score
EMCI: Early mild cognitive impairment
EntCtx: Entorhinal cortex
Frontal: Frontal lobe
GlycA: Glycoprotein acetyls
HSD: Honestly significant difference
Hippo: Hippocampus
LMCI: Late mild cognitive impairment
MEM: Memory composite score
MMSE: Mini-Mental State Exam
MRI: Magnetic resonance imaging
PET: Positron emission tomography imaging
SMC: Significant memory concern
